# Colchester Medical Society

**Published:** 1802-06

**Authors:** 


### *To the Editors of the Medical and Phyſical Journal.*

Gentlemen,

As we perceive you are continuing to give Teſtimonies in ſupport of the Vaccine Inoculation, we have tranſmitted you a copy of one that was forwarded to Dr. Jenner, from the Colcheſter Medical Society, in November laſt; and by him laid before the Committee of the Houſe of Commons; and wiſh, if it meets your approbation, it may find a place in your uſeful Journal.

---

### COLCHESTER MEDICAL SOCIETY.

Eſtabliſhed A. D. 1774.

We the underſigned Members of the Colcheſter Medical Society, having for ſome time practiſed the Vaccine Inoculation with uninterrupted ſucceſs, and ſo fully ſatisfied of the great importance of the diſcovery thereof to mankind, and its efficacy in ſecuring the habit againſt the infection of that dreadful and frequently fatal diſeaſe, the Small-pox, as not only to have recommended it generally in our practice, but to have adopted it in ſuch parts of our own families as have not had that diſeaſe; feel it incumbent upon ourſelves, to teſtify our gratitude tude for the exertions you have with ſuch ſteady perſeverance made in endeavouring to eradicate one of the ſevereſt ſcourges of the human race.

Our ſanguine hopes are, that time will in an equal degree tend to eſtabliſh your reputation and extinguiſh this baneful malady, and that generations to be born not long hence will be as ignorant of its effects as the ancients were of its name.

To you therefore we readily pay ſuch thanks as are due to *one*, who, directed by "the Giver of all good gifts," has been the inſtrument of conferring upon mankind ſo important a benefit. If in the expreſſion of theſe thanks we have not been ſo early as ſome, let it not be imagined that our gratitude is leſs prompt, or our ſenſe of the advantage derived to ſociety leſs ſtrong, but rather, that we wiſhed for the ſanction of time and experience to ratify the Declaration, which we have now the honor to tranſmit to you.

R. R. Newell,

P. Gretton,

J. Godfrey,

T. C. Harrold,

H. Nunn,

J. Hopkins,

